# The monothiol glutaredoxin Grx4 influences thermotolerance, cell wall integrity, and Mpk1 signaling in *Cryptococcus neoformans*

**DOI:** 10.1093/g3journal/jkab322

**Published:** 2021-09-07

**Authors:** Guanggan Hu, Linda Horianopoulos, Eddy Sánchez-León, Mélissa Caza, Wonhee Jung, James W Kronstad

**Affiliations:** Michael Smith Laboratories, Department of Microbiology & Immunology, University of British Columbia, Vancouver, BC V6T 1Z4, Canada; Michael Smith Laboratories, Department of Microbiology & Immunology, University of British Columbia, Vancouver, BC V6T 1Z4, Canada; Michael Smith Laboratories, Department of Microbiology & Immunology, University of British Columbia, Vancouver, BC V6T 1Z4, Canada; Michael Smith Laboratories, Department of Microbiology & Immunology, University of British Columbia, Vancouver, BC V6T 1Z4, Canada; Department of Systems Biotechnology, Chung-Ang University, Anseong 17546, Republic of Korea; Michael Smith Laboratories, Department of Microbiology & Immunology, University of British Columbia, Vancouver, BC V6T 1Z4, Canada

**Keywords:** fungal pathogenesis, stress response, phosphorylation, calcineurin

## Abstract

Monothiol glutaredoxins are important regulators of iron homeostasis that play conserved roles in the sensing and trafficking of iron-sulfur clusters. We previously characterized the role of the monothiol glutaredoxin Grx4 in iron homeostasis, the interaction with the iron regulator Cir1, and virulence in *Cryptococcus neoformans*. This important fungal pathogen causes cryptococcal meningoencephalitis in immunocompromised individuals worldwide. Here, we demonstrate that Grx4 is required for proliferation at elevated temperatures (both 37°C and 39°C) and under stress conditions. In particular, the *grx4Δ* mutant was hypersensitive to SDS, calcofluor white (CFW), and caffeine, suggesting that Grx4 is required for membrane and cell wall integrity (CWI). In this context, we found that Grx4 regulated the phosphorylation of the Mpk1 mitogen-activated protein kinase (MAPK) of the CWI pathway in cells grown at elevated temperature or upon treatment with CFW, caffeine, or SDS. The *grx4Δ* mutant also displayed increased sensitivity to FK506 and cyclosporin A, two inhibitors of the calcineurin pathway, indicating that Grx4 may influence growth at higher temperatures in parallel with calcineurin signaling. Upon thermal stress or calcium treatment, loss of Grx4 also caused partial mis-localization of Crz1, the transcription factor that is a calcineurin substrate. The phenotypes of the *grx4Δ*, *crz1Δ*, and *cna1Δ* (calcineurin) mutants suggest shared contributions to the regulation of temperature, cell wall, and other stresses. In summary, we show that Grx4 is also a key regulator of the responses to a variety of stress conditions in addition to its roles in iron homeostasis in *C. neoformans.*

## Introduction

The basidiomycetous fungus, *Cryptococcus neoformans*, is an opportunistic pathogen that causes life-threatening meningoencephalitis in immunocompromised people including those with HIV/AIDS. Cryptococcal meningoencephalitis is generally fatal if untreated, and it is estimated that cryptococcosis causes 181,000 deaths globally per year (Park *et al.* 2009; Rajasingham *et al.* 2017). Remarkably, cryptococcal meningitis is thought to account for ∼15% of global HIV/AIDS-related deaths (Rajasingham *et al.* 2017; Okurut *et al.* 2020). The key virulence attributes of *C. neoformans* include the ability to proliferate at mammalian body temperature, the formation of a polysaccharide capsule and cell wall melanin, and the secretion of enzymes such as urease (Altamirano *et al.* 2020). In addition, the ability to sense and adapt to diverse stressful host conditions such as an elevated temperature, oxidative stress, and nutrient limitation (including host nutritional immunity) is essential for the fungus to cause disease (Rutherford *et al.* 2019; Westman *et al.* 2019).

We recently identified the monothiol glutaredoxin Grx4 as a binding partner of Cir1, a master regulator of iron-responsive genes and virulence factor elaboration in *C. neoformans* (Jung *et al.* 2006; Attarian *et al.* 2018). The monothiol glutaredoxins Grx3 and Grx4 are important regulators of iron homeostasis in *Saccharomyces cerevisiae* because of their conserved roles in (2Fe-2S) cluster sensing and trafficking (Gupta and Outten 2020). *Cryptococcus neoformans* possesses a single monothiol glutaredoxin, Grx4, and mutants lacking Grx4 are defective in growth at host temperature, elaboration of the virulence factors capsule and melanin, maintenance of iron homeostasis, and the ability to cause disease in a mouse model of cryptococcosis (Attarian *et al.* 2018). Transcriptional profiling by RNA-Seq further revealed a role for the GRX domain of Grx4 in iron homeostasis through the regulation of functions for Fe-S cluster binding, heme biosynthesis, mitochondrial activities, and iron binding and uptake (Attarian *et al.* 2018).

As reported here, we have discovered that *grx4Δ* mutants are also defective in responses to various stresses including elevated temperatures and agents that challenge cell wall integrity (CWI). These observations prompted an examination of interactions between Grx4, calcineurin signaling, and the MAP kinase Mpk1 in the CWI pathway. Calcineurin is an important regulator of intracellular calcium homeostasis, and is required for thermotolerance and pathogenesis in many fungal pathogens of animals or plants (Park *et al.* 2019). The protein is a conserved Ca^2+^-calmodulin (CaM) activated serine/threonine-specific protein phosphatase that mediates calcium—dependent signaling in fungi, including *C. neoformans*, *Cryptococcus gattii*, *Candida albicans*, *Aspergillus fumigatus* (Steinbach *et al.* 2007; Reedy *et al.* 2010; Aboobakar *et al.* 2011; Chen *et al.* 2013; Juvvadi *et al.* 2013, 2020; Park *et al.* 2019). Calcineurin consists of a catalytic A subunit (Cna1) and a regulatory B subunit (Cnb1), and is required for growth at 37°C, virulence, and sexual reproduction in *C. neoformans* (Park *et al.* 2016, 2019). The structure of calcineurin is maintained by two intracellular chaperones, cyclophilin A and FKBP12. Two immunosuppressive natural products, cyclosporine (CsA) and tacrolimus (FK506), bind to cyclophilin A and FKBP12, respectively, and the protein-drug complexes then inhibit calcineurin (Park *et al.* 2019). In response to stress-derived signals, intracellular Ca^2+^ levels increase, the ion binds to calmodulin, and Ca^2+^-calmodulin then binds to Cna1, leading to calcineurin activation. Activated calcineurin dephosphorylates target proteins, which in turn modulate various biological processes. In fungi, the transcription factor Crz1/Sp1 is a major substrate of calcineurin activation, and this factor regulates the transcription of genes involved in stress responses (Stathopoulos-Gerontides *et al.* 1999; Chow *et al.* 2017). In *C. neoformans*, Crz1 plays a role in CWI, induction of autophagy, the response to stress and virulence (Adler *et al.* 2011; Lev *et al.* 2012; Chow *et al.* 2017; Park *et al.* 2016).

*Cryptococcus* *neoformans* also has a PKC/Mpk1 MAP kinase signaling pathway to sense and respond to unfavorable environmental conditions (Kraus *et al.* 2003; Gerik *et al.* 2005, 2008; Wang *et al.* 2011; Lam *et al.* 2013). PKC/Mpk1 signaling is indispensable for thermotolerance, the response to oxidative, nitrosative and cell wall stresses, the elaboration of capsule and melanin, and virulence (Kraus *et al.* 2003; Gerik *et al.* 2005, 2008; Wang *et al.* 2011; Lam *et al.* 2013; Esher *et al.* 2018). The major components of the signaling pathway include the small GTP-binding protein Rho1, Pkc1, Bck1 (MAPKKK), Mkk1/Mkk2 (MAPKK), and the downstream target Mpk1 (Slt2) (MAPK) (Dichtl *et al.* 2016). Activation of Mpk1 by phosphorylation regulates downstream cellular processes, and it is known that calcineurin and Mpk1 play complementing roles in regulating cell integrity in *C. neoformans* (Kraus *et al.* 2003).

In this study, we further examined the growth defects of *grx4Δ* deletion mutants at elevated temperatures (37°C and 39°C), and found that Grx4 influences the phosphorylation of Mpk1 under a variety of stress conditions. We also demonstrated that loss of *GRX4* led to increased susceptibility to the calcineurin-specific inhibitors, FK506 and CsA. Loss of *GRX4* also caused similar phenotypes to the mutants defective calcineurin signaling, including impaired growth with agents that challenge CWI or cause hyperosmotic or endoplasmic reticulum (ER) stress. However, analysis of *grx4Δ* *cna1Δ* double mutants suggested that Grx4 plays a role in parallel with calcineurin signaling in the regulation of thermotolerance and CWI. The *grx4Δ* mutant also shared phenotypes with a *crz1Δ* mutant and Grx4 influenced the nuclear localization of Crz1 in response to stress. Overall, these studies extend the contribution of Grx4 beyond iron homeostasis to include resistance to stress in *C. neoformans*.

## Materials and methods

### Strains, plasmids, and media

Strain H99 of *C. neoformans* var*. grubii* (serotype A) and derived mutants were used in the majority of the experiments. An additional wild-type strain KN99 and the *crz1Δ* mutant in this strain (designated *crz1-MH*) were also used (Liu *et al.* 2008; Chun and Madhani 2010). Strains were maintained on YPD rich medium (1% yeast extract, 2% peptone, 2% dextrose, and 2% agar). Plasmids pCH233, pJAF15, and pJAF1 were the sources of the nourseothricin (NAT), hygromycin (HYG), or neomycin (NEO) resistance cassettes, respectively. YPD plates containing nourseothricin (100 µg/ml), G418 (200 µg/ml), or hygromycin (200 µg/ml) were used to select transformants. All chemicals were from Sigma-Aldrich (St. Louis, MO, USA) unless otherwise stated. Assays on solid media for assessing iron-associated phenotypes employed yeast nitrogen base (YNB) (Difco) supplemented with the iron chelator, bathophenanthroline disulphonate (BPS), adjusted to pH 7.0 with 1M 3-(N-Morpholino) propanesulfonic acid (MOPS). Iron-chelated dH_2_O was prepared by passage of dH_2_O through a column containing Chelex-100 resin (BIORAD Chelex-100) and used in the preparation of YNB-BPS (Jung *et al.* 2009). Defined low-iron media (LIM) [0.5% glucose, 38 mM L-asparagine, 2.3 mM K_2_HPO_4_, 1.7 mM CaCl_2_·2H_2_O, 0.3 mM MgSO_4_·7H_2_O, 20 mM HEPES, 22 mM NaHCO_3_, 1 ml of 1000X salt solution (0.005 g/L CuSO_4_·5H_2_O, 2 g/L ZnSO_4_·7H_2_O, 0.01 g/L MnCl_3_·4H_2_O, 0.46 g/L sodium molybdate, 0.057 g/L boric acid), in iron-chelated dH_2_O adjusted to pH 7.4 with 0.4 mg/L sterile thiamine added post-filtering] was prepared as described previously (Lian *et al.* 2005; Tangen *et al.* 2007).

### Construction of gene deletion mutants

The *cna1Δ*, *cna1Δ* *grx4Δ*, *crz1Δ*, and *crz1Δ* *grx4Δ* deletion mutants were constructed by homologous recombination using gene-specific knockout cassettes assembled by three-step overlapping PCR (Hu *et al.* 2008) with the primers listed in Supplementary Table S1. The resistance markers NEO, NAT, and HYG were amplified by PCR using primers 2 and 5 and the plasmids pJAF1, pCH233, and pJAF15, respectively, as the templates. In general, the gene-specific knockout primers 1 and 3 and 4 and 6 were used to amplify the flanking sequences of their respective genes; and primers 1 and 6 were used to amplify the gene-specific deletion construct containing the resistance marker. All constructs for deletions were introduced into the H99 WT strain (for generation of *cna1Δ* and *crz1Δ* mutants) (Caza *et al.* 2018), or a *grx4Δ* mutant (for generation of *grx4Δ* *cna1Δ* double deletion mutants using a *CNA1* deletion construct), or a *crz1Δ* mutant (for generation of *crz1Δ* *grx4Δ* double deletion mutants using a *GRX4* deletion construct), by biolistic transformation, as described previously (Davidson *et al.* 2002). Colony PCR was performed to identify positive transformants using primers 7NE and 8NE, 9PO and NAT3L, and 10PO and NAT5R, respectively; mutant genotypes were confirmed by Southern blot analysis. Two independent mutants of each gene were used for all experiments.

To generate the strain expressing Mpk1-FLAG in the H99 background, a PCR product was amplified from JLCN554 (KN99 background, provided by Dr. Jennifer Lodge) using primer pair Mpk1-FLAG-P1F/Mpk1-FLAG-P3R (Supplementary Table S1), and biolistically transformed into the WT (H99) strain with selection on G418 (200 µg/ml). The resulting transformants were screened and confirmed by PCR, examined phenotypically, and found to behave like the WT (H99) strain. The *grx4Δ* deletion construct was amplified from the *grx4Δ* mutant, and transformed into the Mpk1-Flag strain (H99 background) to generate the *grx4Δ* -Mpk1-Flag strains. The *grx4Δ* *-*Mpk1-Flag strains were confirmed by PCR and examined for *grx4Δ*-specific phenotypes. The *grx4Δ* -Mpk1-Flag strain showed similar phenotypes to the *grx4Δ* mutant described previously (Attarian *et al.* 2018).

### Capsule formation

Formation of the polysaccharide capsule and cell morphology were examined by differential interference microscopy (DIC) on an Axioplan 2 imaging microscope (Zeiss), after incubation for 24 h at 30°C in defined low-iron medium (LIM) and staining with India ink.

### Stress and drug response assays

To examine the responses of the WT, *grx4Δ, cna1Δ, crz1Δ*, and *grx4Δ* *cna1Δ* and *crz1Δ* *grx4Δ* strains to various stress conditions, cells from exponentially growing cultures were washed, resuspended in H_2_O, and adjusted to a concentration of 2 × 10^4^ cells per milliliter. The cell suspensions were diluted 10-fold serially, and 5 μl of each dilution was spotted onto YPD and/or YNB plates supplemented with different compounds. Plates were incubated for 2–10 days at 30°C, 37°C, or 39°C, and photographed. The specific assays were performed on YPD and/or YNB plates supplemented with 1.2 M KCl, 1.2 M NaCl, 100 mM LiCl, 0.01% or 0.02% SDS, 0.5 mg/ml Congo Red, 0.5 mg/ml or 1.5 mg/ml caffeine, or 0.65 mg/ml calcofluor white (CFW). Sensitivity to the inhibitors brefeldin A (BFA) and monensin was examined by spotting the cell dilutions on YPD containing 30 μg/ml of BFA or 625 μg/ml of monensin. Osmotic rescue of mutant growth was performed by spotting cells onto YPD plates with 1M sorbitol.

### Protein extraction and immunoblot analysis

The cells were grown overnight at room temperature (25°C) to late logarithmic phase in 50 ml of YPD medium, diluted 1 in 10 in fresh YPD and grown in a final volume of 50 ml for 4 h at 25°C with shaking. For thermal stress treatment, the cells were diluted 1:1 with YPD medium prewarmed at 55°C and further incubated at 39°C for the indicated time intervals. Control cells were diluted 1:1 with room temperature YPD and incubated further at 25°C. Protein extracts were obtained with a modified lysis buffer (50 mM Tris-HCl pH7.5, 5 mM EDTA, 100 mM NaCl, 1% Triton X-100, and 1× EDTA-free protease inhibitor cocktail (Roche, Basel, Switzerland). Protein concentration was determined using the Pierce™ BCA Protein Assay kit following the manufacturer’s instructions (Thermo Fisher, Waltham, MA, USA). For all immunoblot analyses, proteins were transferred onto PVDF (GE Healthcare, Boston, MA, USA) using a wet transfer at 80 V for 1 h. Membranes were blocked in Tris-buffered saline with Tween 20 (TBST) with 5% skim milk and incubated with the following antibodies at the indicated concentrations: monoclonal anti-FLAG (Thermo Fisher) at 1:5000, anti-phosphorylated Mpk1(Slt2) (Cell Signaling Technology), anti-histone H3 (Millipore Sigma), anti-beta actin (Genescript), and anti-mouse HRP (Bio-Rad, Hercules, CA, USA) at 1:5000. Immunoblots were visualized using chemiluminescence (GE Healthcare), and the signals were quantified using the ImageJ program.

### Real-time PCR

Real-time PCR analysis was conducted as previously described (Hu *et al.* 2007, 2015) using primers designed with Primer Express (Applied Biosystems, http://www.appliedbiosystems.com). Briefly, total RNA from frozen cells was extracted the using the RNeasy mini kit (Qiagen), DNA was removed by treatment with Turbo Dnase (Ambion) for 30 min at 25°C, and cDNA was synthesized using a mixture of anchored oligo dT random hexamers (3:1) and Superscript transcriptase II (Invitrogen, Canada). The resulting cDNA was used for real-time PCR with qPCR SYBR Green master mix (Bio Basic) according to manufacturer’s recommendations. An Applied Biosystems 7500 Fast Real-Time PCR System was used to detect and quantify the PCR products using the following conditions: incubation at 95°C for 10 min followed by 40 cycles of 95°C for 15 s and 60°C for 1 min. The cDNA of the actin gene and/or 18S RNA was used to normalize the data. Dissociation analysis on all PCRs confirmed the amplification of a single product for each primer pair and the lack of primer dimerization (Applied Biosystems). The primers for each gene are listed in Supplementary Table S2. Relative gene expression was quantified using SDS software 2.3.1 (Applied Biosystems) and the 2-ΔΔCt method (Livak and Schmittgen 2001).

### Flow cytometry

Fungal cells were grown in YNB overnight at 30°C, 37°C, or 39°C, respectively. The cells were diluted to OD_600_ = 1 and stained for chitin or chitosan with 100 µg/ml CFW in PBS pH 7.4 or 250 µg/ml Eosin Y (EoY) in McIllvaine’s buffer pH 6.0, respectively. After the staining, cells were washed with buffer as previously described (Santiago-Tirado *et al.* 2015; Horianopoulos *et al.* 2020). All flow cytometry data were collected on an Attune Nxt flow cytometer (Invitrogen). The BL1 filter was used with eosin Y dye and the VL1 filter with CFW. Flow cytometry data were analyzed using FlowJo v10 software (FlowJo, LLC, Ashland, OR, USA), and statistical significance was evaluated by performing ANOVAs with Tukey’s multiple comparisons in GraphPad Prism v7 (GraphPad Software).

### Fluorescence microscopy

Plasmid pEC13 carrying the gene fusion for Crz1-mCherry was provided by Dr. Joe Heitman (Chow *et al.* 2017), and the PacI—linearized plasmid DNA was biolistically transformed into a *crz1Δ* deletion mutant generated in a previous study (Caza *et al.* 2018). The resulting strain (*crz1Δ* + *CRZ1*-mCherry) complemented the *crz1Δ* phenotypes (Caza *et al.* 2018, G.H. and M.C., unpublished results). Deletion of *GRX4* was performed by biolistically transforming the *grx4Δ* deletion construct into the *crz1Δ* + *CRZ1*-mCherry strain, as previously described (Attarian *et al.* 2018), resulting a *grx4Δ* Crz1-mCherry strain. For fluorescence microscopy, cells were grown in YPD overnight at the indicated temperatures, fixed with 4% formaldehyde for 15 min, washed in PBS, and stained with DAPI (4’,6’-diamidino-2-phenylindole, 1 µg/ml, for staining nuclei) before microscopic observation. To test the influence of CaCl_2_ and low iron on Crz1-mCherry localization, the cells were grown at 25°C with shaking overnight, washed, and diluted in PBS. Cells were then transferred into fresh liquid YPD supplemented with or without 200 mM CaCl_2_ or into low-iron YNB medium supplemented with 150 µM BPS (bathophenanthroline disulphonate, iron chelator) and incubated at 25°C for additional 60 min before microscopic observation. Cells were examined with fluorescence microscopy on an Axioplan 2 imaging microscope (Zeiss) with magnification 1000×, and Zen Lite software.

### Inductively coupled plasma–atomic emission spectroscopy (ICP–AES)

ICP–AES was employed to measure the total cellular calcium and iron in the wild-type strain H99, and the *grx4Δ* and *cir1Δ* mutants. The strains were grown in 50 ml of YPD medium at 30°C overnight, and cells were washed twice with low-iron water and resuspended in 200 ml of defined low-iron YNB medium followed by incubation at 30°C for 2 days. Cells were washed three times with low-iron water and lyophilized. A total of 0.15 g of cell biomass was digested with 3 ml of H_2_O_2_ and 5 ml of HNO_3_ using a microwave digestion system (START D). ICP–AES analysis was performed using the OPTIMA 5300 DV (PerkinElmer) system. The scaling and normalization process were based on total cell mass.

## Results

### Mutants lacking Grx4 display temperature sensitivity and compromised cell wall integrity

**Figure 1:**
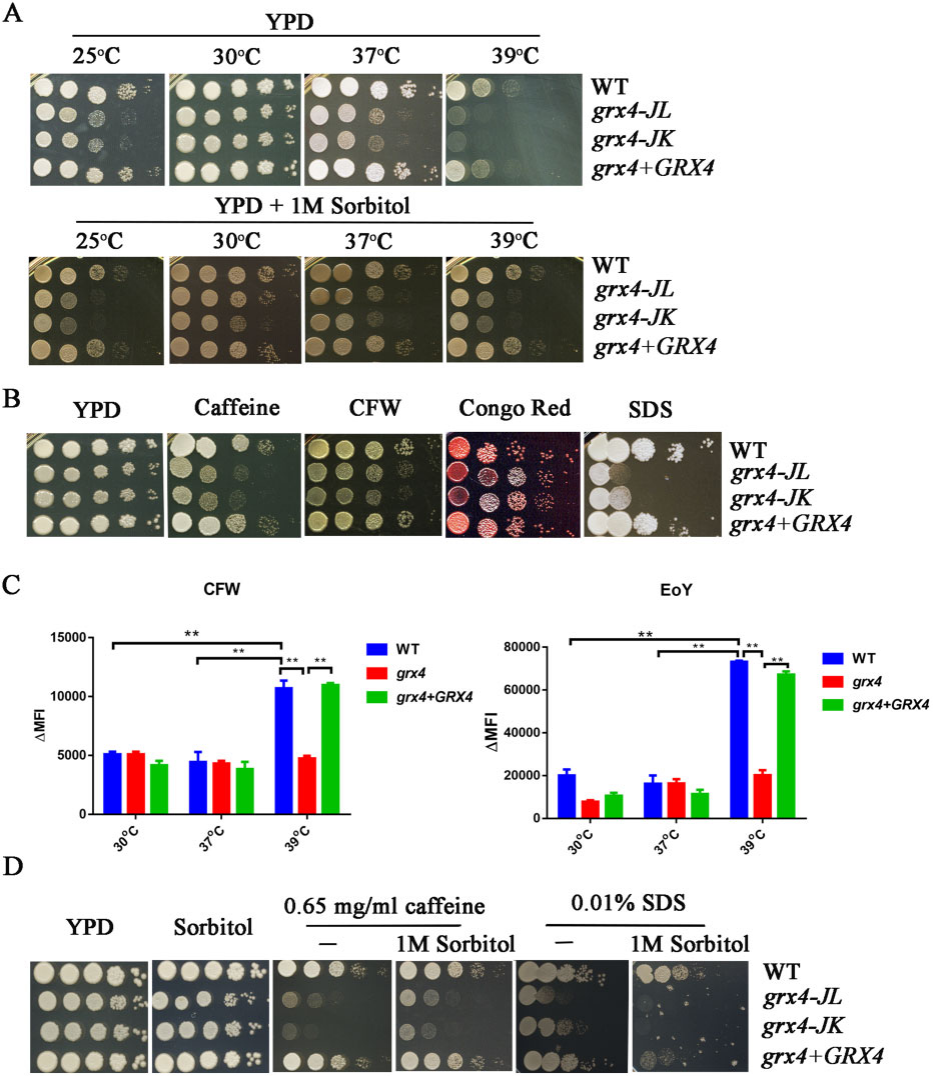
Loss of Grx4 impairs thermotolerance and cell wall integrity. (A) Cells of the WT strain, two independent *grx4Δ* mutants [labeled *grx4-JL* and *grx4-JK* (Attarian *et al.* 2018)] and the *GRX4* complemented strain were grown overnight at 30°C. Ten-fold serial dilution of the cells were spotted onto YPD with or without sorbitol followed by incubation for 2 days at the indicated temperatures before being photographed. (B) Cells of the same strains were spotted on YPD supplemented with or without either 0.65 mg/ml caffeine, 0.65 mg/ml CFW, 0.5 mg/ml Congo Red, or 0.01% SDS, and incubated at 30°C for 2 days before photographed. (C) Cells of the strains were grown in liquid YNB medium at either 30°C, 37°C, or 39°C overnight, washed with buffer, and stained with 100 µg/ml CFW to detect exposed chitin or 250 µg/ml eosin Y (EoY) to detect chitosan. The fluorescence intensity of the cells was measured by flow cytometry and analyzed by FloJo v10. ΔMFI = change in mean fluorescence intensity. (D) The strains were grown overnight at 30°C and spotted onto the YPD plates supplemented with sorbitol alone or sorbitol with or without caffeine or SDS, and the plates were incubated at 30°C for 2 days before being photographed.

Previously, we observed that Grx4 is required for robust growth at 37°C and in low iron conditions, and for virulence in a mouse model of cryptococcosis (Attarian *et al.* 2018). In this study, we initially extended our analysis to test the growth of the *grx4Δ* mutant at lower (25°C) and higher (39°C) temperatures (Figure 1). In comparison to the WT level of growth at 30°C, and the reduced growth at 37°C observed previously, we found a more severe growth impairment for the *grx4Δ* mutants at 39°C as well as delayed growth at room temperature (25°C) after 2 days. However, there was no significant difference at 25°C after 5 days of incubation compared with the WT strain thus indicating a delayed growth phenotype (Figure 1A and G.H., unpublished results). The *GRX4* reconstituted strain restored growth to the WT level at all temperatures examined. Overall, the results indicate a growth defect for the *grx4Δ* mutant in sub-optimal temperature conditions, with a particular impact at the elevated temperature of 39°C.

The temperature sensitivity of the *grx4Δ* mutant prompted us to examine the ability of the mutant to respond to additional stresses including agents that challenge membrane and CWI. We therefore spotted cells of the WT strain, the *grx4Δ* mutants and the complemented strain onto YPD media supplemented with SDS, Congo Red, caffeine, or CFW. The detergent SDS disrupts the plasma membrane, whereas CFW and Congo Red interfere with the synthesis of cell wall polysaccharides (Elorza *et al.* 1983; Wood and Fulcher 1983; Dichtl *et al.* 2016; Liu *et al.* 2021). Caffeine has a variety of targets in fungi including target of rapamycin (TOR) signaling and components of the CWI pathway (Ruta and Farcasanu 2020). The *grx4Δ* mutants exhibited increased sensitivity to SDS, caffeine and CFW indicating that Grx4 is required for membrane and CWI; the Grx4 reconstituted strain restored growth to the WT level (Figure 1). Interestingly, the *grx4Δ* mutants grew normally on medium with Congo Red, although the colonies appeared darker in color suggesting an altered interaction with the dye.

To carry out a more detailed analysis of the cell wall, we examined WT, *grx4Δ* and *GRX4* complemented strains by staining chitin on the cells with CFW and chitosan with Eosin Y, and performing flow cytometry. In this assay, cells were grown overnight in YNB at 30°C, 37°C, or 39°C to assess the influence of temperature. No differences in fluorescence intensity were observed for cells grown at 30°C or 37°C suggesting no significant alteration in exposed chitin or chitosan between the strains (Figure 1C). In contrast, lower fluorescence intensities indicative of reduced exposed chitin and chitosan were observed for the *grx4Δ* mutant compared to the other strains grown at 39°C; similar levels of exposed chitin and chitosan were found for the WT and complemented strains. It was notable that the changes in mean fluorescence intensities at 39°C were markedly higher than at 30°C or 37°C in all examined strains, indicating an impact of elevated temperature on cell wall structure. In general, loss of Grx4 resulted in changes in cell wall architecture under thermal stress, and this may partially explain the defective growth of *grx4Δ* mutants at elevated temperatures and with inhibitors of CWI.

Sorbitol is an osmotic stabilizer that can rescue the growth of mutants with defects in CWI (e.g., mutants lacking Mpk1) in *C. neoformans* (Kraus *et al.* 2003). We therefore examined whether 1M sorbitol could improve the growth of *grx4Δ* mutants incubated at the different temperatures and found partial rescue at both 37°C and 39°C (Figure 1A). We also tested whether sorbitol would rescue the growth of *grx4Δ* mutants on YPD plates supplemented with 0.65 mg/ml of caffeine or 0.01% SDS (Figure 1D). Interestingly, sorbitol partially restored the growth of the *grx4Δ* mutants on caffeine but actually exacerbated the sensitivity of the strains to SDS, likely as a result of increased osmotic stress on the plasma membrane (Figure 1D). Taken together, these results generally support a connection between Grx4, thermotolerance, and membrane/CWI .

### Loss of Grx4 confers sensitivity to multiple stress conditions

**Figure 2:**
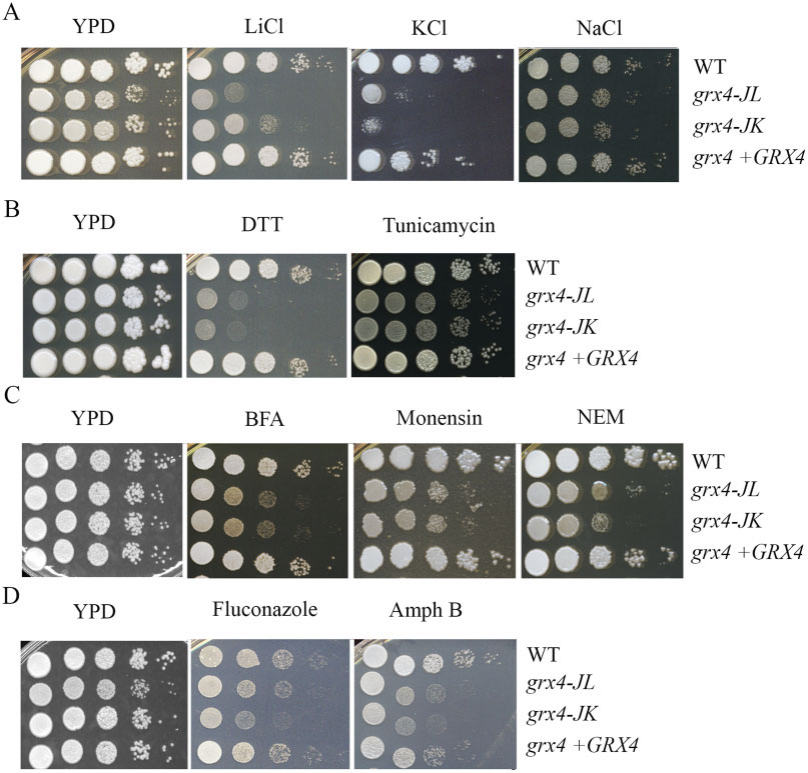
Grx4 is required for resistance to salt and ER stress, trafficking inhibitors, and antifungal drugs. (A) Cells of the indicated strains were grown overnight at 30°C, and 10-fold serial dilutions were spotted onto YPD with or without the following supplements: (A) 75 mM LiCl, 1.5M KCl or 1.5M NaCl.; (B) 15 mM DTT or 250 ng/ml tunicamycin; (C) 40 µg/ml brefeldin A (BFA); 0.5 mg/ml monensin, or 100 µM NEM; (D) 10 µg/ml fluconazole or 0.5 mg/ml amphotericin B (Amph B). All plates were incubated at 30°C for 2 days before being photographed.

We previously demonstrated that Grx4 plays a role in counteracting oxidative stress in *C. neoformans* (Attarian *et al.* 2018), and we hypothesized that the protein may play a broader role in the response to stress. We therefore tested mutant growth in several additional stress conditions. Initially, we found that deletion of *GRX4* resulted in reduced growth on solid media supplemented with NaCl, KCl, or LiCl indicating a role in the response to ionic stress (Figure 2A). Similarly, the *grx4Δ* mutants showed greater sensitivity to the ER stress reagents, dithiothreitol (DTT), and tunicamycin (Figure 2B). DTT is a reducing agent targeting disulfide bonds of proteins to induce proteotoxic stress and tunicamycin is an inhibitor of glycoprotein synthesis inducing the unfolded protein response in the ER. Our observations therefore implicate Grx4 in the response to ER stress, and we extended our analysis to examine the connection between *GRX4* and intracellular trafficking, in particular, ER-Golgi transport. Specifically, we tested the growth of the *grx4Δ* mutants on YPD plates supplemented with the trafficking inhibitors brefeldin A (BFA), monensin, or N-ethylmaleimide (NEM) (Figure 2C). BFA is a reagent that arrest the anterograde transport of proteins between the ER and the Golgi compartment, and monensin is a Na^+^/H^+^ ionophore that blocks intracellular transport in both the trans-Golgi and post-Golgi compartments. NEM is cysteine alkylating agent that interferes with disulfide bond formation in proteins that blocks vesicular transport. Interestingly, loss of *GRX4* caused increased sensitivity to each of the inhibitors (Figure 2C) thus indicating a role not only in response to ER stress but also ER-Golgi intracellular trafficking. Finally, we found that the *grx4Δ* mutants displayed increase susceptibility to fluconazole and amphotericin B, two antifungal drugs used clinically to treat cryptococcosis (Figure 2D). Fluconazole inhibits ergosterol biosynthesis in the ER and amphotericin B acts at the plasma membrane. The sensitivity of the mutants is therefore consistent with a role for Grx4 in maintaining membrane integrity. In all conditions, the *GRX4* reconstituted strain restored the growth to the WT level.

### Grx4 regulates the phosphorylation of Mpk1 in response to stress

**Figure 3:**
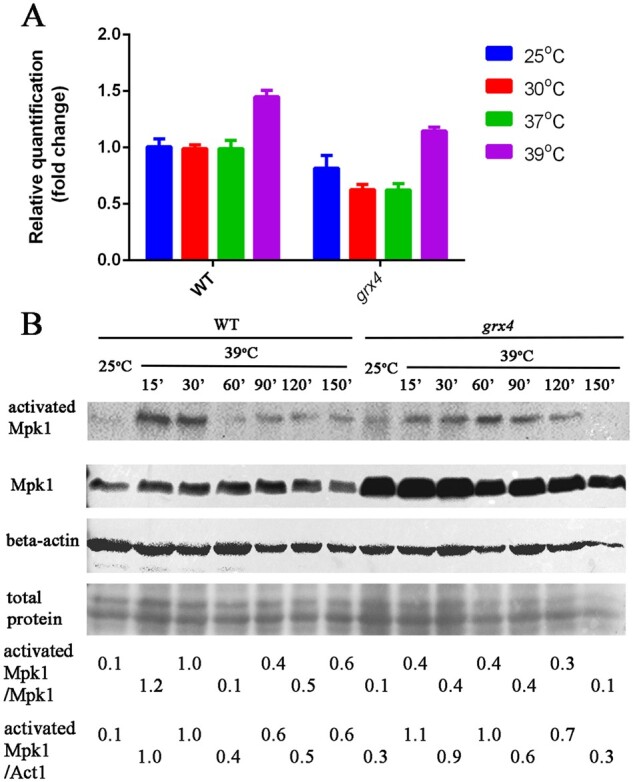
*MPK1* transcript levels are upregulated at 39°C, and Mpk1 phosphorylation is dependent on Grx4. (A) Cells of WT and the *grx4Δ* mutant (labeled *grx4*) from overnight cultures in YPD were transferred into fresh YPD medium and incubated for 30 min at the indicated temperature before RNA isolation. Transcript levels for *MPK1* were measured by qRT-PCR; the Y-axis indicates the abundance of the *MPK1* transcripts in the different strains and conditions relative to the WT strain at 25°C. Each bar represents the average and standard deviation for three biological replicates, and statistically significant differences relative to the transcript levels in WT cells at 25°C were determined by an unpaired *t*-test in Graphpad Prism v7. (B) Immunoblot analysis to detect Mpk1 protein level and phosphorylation status for the WT strain or the *grx4Δ* mutant grown at 25°C or 39°C. The strains were grown at 25°C overnight, transferred to 39°C, and harvested at the indicated time points. The noninduced control for each strain was harvested before transfer of the cells to 39°C. The phosphorylation status of Mpk1 was monitored using a rabbit phosphor p-42/44 antibody (activated Mpk1). The same blot was re-stripped and probed with an anti-FLAG antibody to detect total Mpk1. The blots were re-stripped further and probed with mouse beta-actin antibody as a control for protein loading. The numbers at the bottom indicate the ratio of the signals for the activated Mpk1 and total Mpk1 (all treatments), or for the activated Mpk1 and actin protein as determined ImageJ analysis of the scanned images.

Given the contribution of Grx4 to stress tolerance, we hypothesized that the protein influenced key stress pathways such as the CWI pathway (Dichtl *et al.* 2016). This idea is supported by the finding that the monothiol glutaredoxins Grx3 and Grx4 physically interact with the MAP kinase Slt2 to participate in the response to oxidative stress in *S. cerevisiae* (Pujol-Carrion *et al.* 2013; Pujol-Carrion and Torre-Ruiz 2017). In *C. neoformans* and other fungi, the ortholog of Slt2, Mpk1, is the terminal MAP kinase in the mitogen-activated protein (MAP) kinase cascade of the CWI pathway (Dichtl *et al.* 2016). The CWI pathway is well characterized in *C. neoformans* and influences elaboration of capsule and melanin, growth at elevated temperature, CWI, and the responses to oxidative stress and antifungal drugs (Chang and Penoyer 2000; Kraus *et al.* 2003; Gerik *et al.* 2008; Wang *et al.* 2011; Lam *et al.* 2013; Donlin *et al.* 2014). To examine whether Grx4 regulates thermotolerance and CWI via the CWI pathway, we first measured the transcripts of *MPK1* by qRT-PCR in both WT and *grx4Δ* mutant cells grown at different temperatures (Figure 3A). There was no significant difference in *MPK1* transcript levels between 25°C, 30°C, or 37°C in the WT strain or in the *grx4Δ* mutant, and increased levels of *MPK1* transcripts were observed in cells of both strains grown at 39°C.

The CWI pathway is activated at elevated temperature in fungi including *S. cerevisiae* and *C. neoformans* (Kamada *et al.* 1995; Lam *et al.* 2013: Dichtl *et al.* 2016). We therefore tested the kinetics of Mpk1 activation by measuring phosphorylation of the protein in the WT strain and the *grx4Δ* mutant. The Mpk1 protein was fused with a FLAG tag, and the strains expressing the fusion had WT phenotypes on stress conditions indicating that the protein was functional (G.H., unpublished results). By immunoblotting with an antibody specific for phosphorylated Mpk1, we first verified that thermal stress at 39°C triggered Mpk1 phosphorylation, as described by others (Lam *et al.* 2013). As expected, an increase in Mpk1 phosphorylation was observed after WT cells were incubated at 39°C compared to cells incubated at 25°C with a peak at 15 and 30 min (Figure 3B). In contrast, Mpk1 phosphorylation was reduced in cells of the *grx4Δ* mutant subjected to 39°C, with a weak signal of phosphorylation at early times and a later peak at 60 min. The total amount of Mpk1 protein, detected using an anti-Flag antibody, did not change when WT or mutant cells were incubated at 39°C compared to 25°C, but loss of Grx4 resulted in an increase in Mpk1 protein at both 25°C and 39°C, compared with the WT strain (Figure 3B). Given the lack of a marked influence of Grx4 on *MPK1* transcript levels (Figure 3A), the protein may have a post-transcriptional influence on the level of Mpk1 protein. Overall, these results suggest that Grx4 regulates thermotolerance at least in part by influencing that activation of Mpk1 in the CWI pathway.

**Figure 4:**
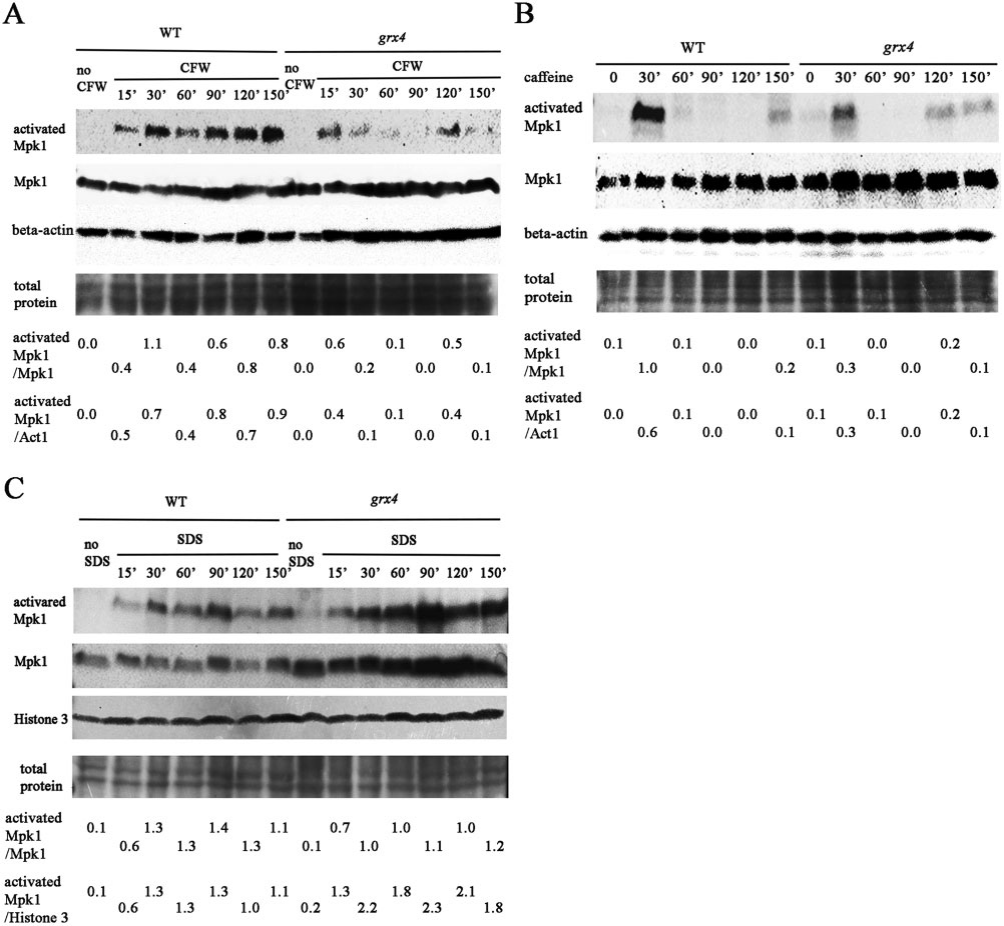
Grx4 influences Mpk1 phosphorylation in response to CFW, caffeine, and SDS. Cells of the WT strain and the *grx4Δ* mutant (labeled *grx4*) were grown overnight in YPD and transferred to fresh medium supplemented with (A) 20 µg/ml CFW; (B) 0.5 mg/ml caffeine or; (C) 0.01% SDS. The cells were incubated for the indicated times before protein extraction. Phosphorylation of Mpk1 and total Mpk1 protein were analyzed as in Figure 3, except that anti-histone 3 antibody was used to assess equal loading in C. The numbers at the bottom indicate the ratio of the western blot signals for the activated Mpk1 and total Mpk1, or for the activated Mpk1 and histone 3 protein as determined by ImageJ analysis of the scanned images.

We also examined Mpk1 phosphorylation in the WT strain and the *grx4Δ* mutant in response to the cell wall (CFW, caffeine) and membrane (SDS) stressors (Figure 4). In the WT strain treated with CFW, Mpk1 phosphorylation was detected at 15 min and increased up to 150 min (Figure 4A). Mpk1 phosphorylation was also detected at 15 min after the CFW treatment in the *grx4Δ* mutant, but was weaker during incubation up to 150 min. Interestingly, in the WT strain treated with caffeine, a strong signal of Mpk1 phosphorylation was only detected at 30 min, and the signal was weakly discernable at the later times (particularly at 150 min) (Figure 4B). A similar pattern was detected in the *grx4Δ* mutant strain with a somewhat weaker signal at 30 min of caffeine treatment, and an even weaker signal at 120 and 150 min. It is known that caffeine has additional influences on cellular functions beyond an impact on the CWI pathway (Ruta and Farcasanu 2020). A different pattern of phosphorylation was observed upon treatment with SDS (Figure 4C). In this case, prolonged phosphorylation of Mpk1 was seen throughout the experiment for both the WT strain and the *grx4Δ* mutant, but a higher level of phosphorylation was observed in cells of the mutant. Thus, each of the stressors provoked a different pattern of phosphorylation, and Grx4 had different influences including a weaker prolonged response to CFW, an overall weaker response to caffeine, and an elevated and prolonged response to SDS. We also observed the increase in Mpk1 abundance in the *grx4Δ* mutant relative to the WT strain for these treatments, as seen with the response to elevated temperature (Figure 3B). Overall, these observations suggest that Grx4 contributes to thermotolerance, and membrane and CWI, by influencing the abundance and phosphorylation of Mpk1.

### Grx4 mediates stress tolerance in parallel with calcineurin signaling

In addition to the Mpk1/CWI pathway, calcineurin also mediates the response of *C. neoformans* to agents that challenge cell wall and membrane integrity (Park *et al.* 2016; Chow *et al.* 2017; Fu *et al.* 2018). A *C. neoformans* mutant lacking the catalytic subunit Cna1 of calcineurin is hypersensitive to a variety of stress conditions included elevated temperature, oxidative and ionic stress, and chemicals that provoke ER stress (Park *et al.* 2016: Chow *et al.* 2017; Fu *et al.* 2018). Given that the *grx4Δ* mutant shared these phenotypes, we specifically tested the possible interaction of Grx4 and calcineurin by examining calcium levels, the response of the *grx4Δ* mutant to inhibitors of calcineurin, and the phenotypes of *grx4Δ* *cna1Δ* double mutants.

**Figure 5:**
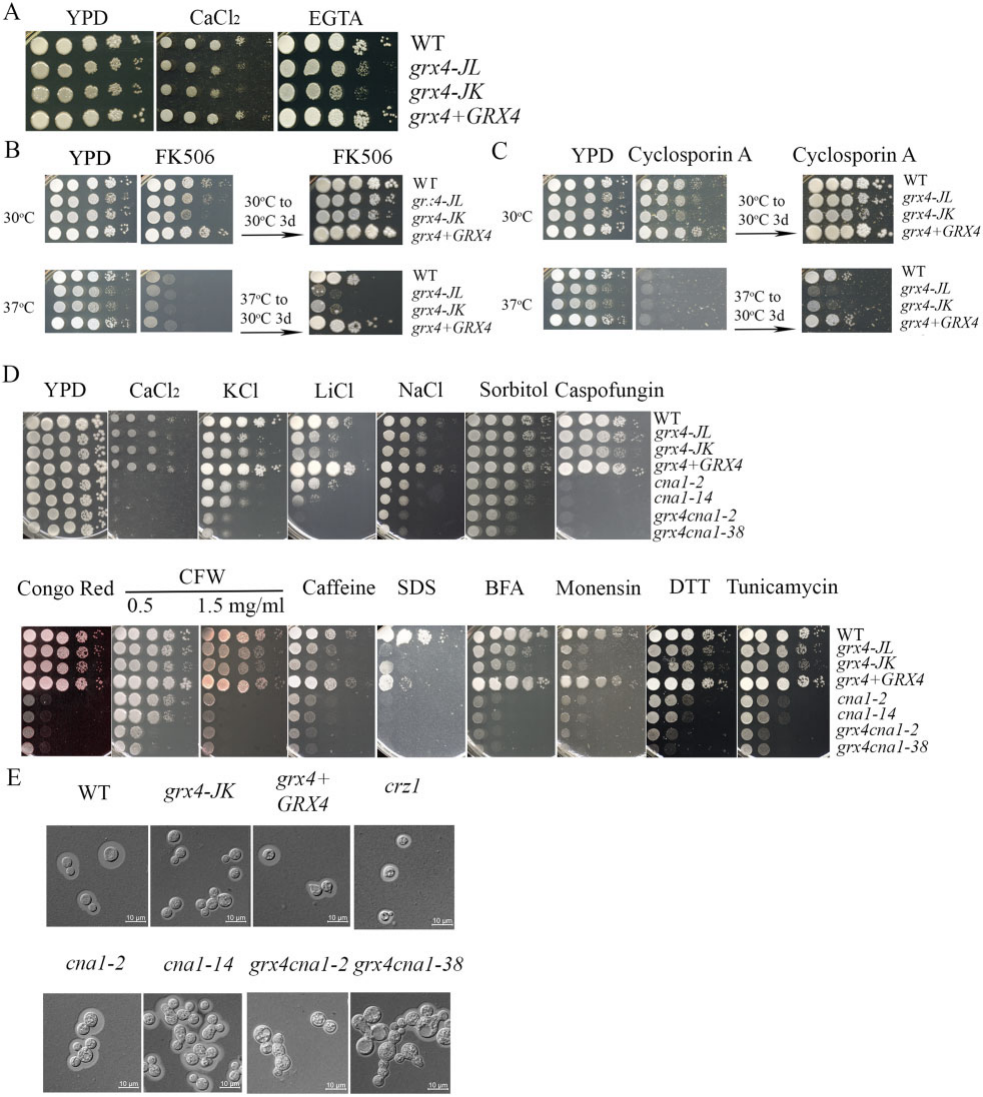
Grx4 and calcineurin subunit Cna1 interact to regulate stress responses. (A) The WT strain, two *grx4Δ* mutants (labeled *grx4-JL* and *grx4-JK*) and the *GRX4* complemented strain were grown overnight at 30°C. Ten-fold serial dilution of cells were spotted on YPD, with or without excess calcium chloride (400 mM) or the calcium chelator EGTA [5 mM, ethylene glycol-bis (β-aminoethyl ether)-N, N, N′,N′-tetraacetic acid], and incubated at 30°C for 2 days before photographed. B) Ten-fold serial dilution of the cells were spotted on YPD supplemented with 1 µg/ml of FK506, and incubated for 2 days at 30°C or 37°C. The plates were then transferred to 30°C for 3 days before photographed. (C) The experiment in (B) was performed in parallel with YPD supplemented with 100 µg/ml of cyclosporin A. (D) Spot assays were also performed with the WT strain, the *grx4Δ* mutants, the *GRX4* complemented strain, two independent *cna1Δ* mutants (labeled *cna1-2* and *cna1-14*), and two independent *grx4Δ cna1Δ* double mutants (labeled *grx4 cna1-2* and *grx4 cna1-38*). YPD was supplemented with 75 mM LiCl, 1.5M KCl or 1.5M NaCl, 1M Sorbitol, 15 mM DTT or 250 ng/ml tunicamycin, 0.5 or 1.5 mg/ml CFW, 0.65 mg/ml caffeine, calcium chloride (400 mM), 0.01% SDS, 40 µg/ml brefeldin A (BFA), or 0.5 mg/ml monensin. The plates were incubated for 2 days before photographed. (E) Photographs of the cellular morphology for the strains tested in D along with a *crz1Δ* mutant (labeled *crz1-hyg*, see Figure 6). The cells were grown in defined low iron medium (capsule inducing medium) overnight at 30°C, stained with India ink, and examined under differential interference contrast microscopy. Bar = 10 µM.

Calcineurin plays a central role in coordinating the cellular responses to Ca^2+^ signaling. We therefore first tested the influence Grx4 in the response to Ca^2+^ by spotting the cells of WT, *grx4Δ* mutant and *GRX4* complemented strains on YPD supplemented with either 400 mM CaCl_2_, or 5 mM of the Ca^2+^ chelator EGTA (Figure 5A). The *grx4Δ* mutant displayed minor growth defects on media with either low or excess Ca^2+^ compared to the WT and complemented strains. We also measured Ca^2+^ levels in the WT strain and the *grx4Δ* and *cir1Δ* mutants, and found that the mutants had perturbed Ca^2+^ levels (Table 1). We performed these measurements on cells grown in with and without iron limitation, given the role of Grx4 in iron homeostasis (Attarian *et al.* 2018). The WT strain contained ∼ 4 times more Ca^2+^ than the *grx4Δ* mutant in the iron-limiting condition, but a higher Ca^2+^ concentration (∼ 4-fold) was noted in the *grx4Δ* mutant *vs* the WT strain in iron rich medium. The WT strain did not show a drastic change in Ca^2+^ levels in response to the two iron conditions. Each of the strains contained a higher level of iron in iron rich media than in iron limiting media, and the *grx4Δ* mutant accumulated more iron than the other strains in both iron rich or limiting conditions. For comparison, we found that the Grx4 interacting partner, Cir1, contained lower concentrations of calcium but higher of iron than in WT, in both iron-limiting and iron-rich conditions (Table 1). Taken together, loss of *GRX4* had a notable impact on Ca^2+^ homeostasis, and iron likely influences the response of Grx4.

**Table 1:** Measurement of calcium and iron concentrations

Strains	Low iron medium		High iron medium	
	Calcium	Iron	Calcium	Iron
WT	63.14 + 0.15	0.4 + 0.06	60.23 + 0.68	258.58 + 3.39
*cir1Δ*	14.68 + 0.06	35.75 + 0.35	24.94 + 0.14	282.96 + 3.64
*grx4Δ*	16.97 + 0.23	4.46 + 0.06	237.35 + 1.81	559.71 + 3.55

Metal contents in the cells were measured by TCP and is presented as µg/g dry cell weight. The experiment was performed three times and standard deviations are presented. The differences in calcium or iron concentrations, between WT and *cir1Δ*, and between WT and *grx4Δ* are significant (*P* < 0.05) in both the low and high iron conditions.

Calcineurin activity is inhibited by the immunosuppressive, antifungal drugs tacrolimus (FK506) and cyclosporine A (CsA), which bind to the immunophilins FKBP12 and cyclophilin A, respectively. We reasoned that the deletion of *GRX4* might cause sensitivity to the calcineurin-specific drugs if the requirement of Grx4 to grow at the elevated temperature was associated with Ca^2+^ triggered calcineurin signaling. We therefore examined the sensitivity of the WT, *grx4Δ*, and *GRX4* reconstituted strains to both FK506 and CsA on YPD medium supplemented with either 1 µg/ml FK506 or 100 µg/ml CsA. At 30°C, two independent *grx4Δ* deletion strains exhibited slightly reduced growth compared to the WT strain at 2 days of incubation, but grew at the WT level after 3 days of further incubation, showing that the sensitivity of cells to either FK506 or CsA was not changed by the *grx4Δ* deletion at 30°C. At 37°C, however, loss of Grx4 resulted in increased susceptibility to both calcineurin-specific inhibitors. After 2 days of incubation at 37°C, all of the strains failed to grow on medium supplemented with either 1 µg/ml FK506 or 100 µg/ml CsA. The agar plates with either FK506 or CsA, which had been incubated for 2 days at 37°C, were then transferred to 30°C for an additional 3 days, and the *grx4Δ* mutants showed marked impaired growth (Figure 5, B and C). In summary, the findings suggest that Grx4 may act in parallel with Ca^2+^ induced calcineurin signaling to regulate the thermotolerance.

To examine the connections between Grx4 and calcineurin in more detail, we deleted the *CNA1* gene in the *grx4* mutant background, and compared the phenotypes of the *grx4Δ*, *cna1Δ*, and *grx4Δ* *cna1Δ* mutants. Two independent mutants for each gene were tested. As expected, the *grx4Δ* or *cna1Δ* single mutants displayed increased sensitivity to ionic, osmotic, and membrane/cell wall stresses, as well as ER and intracellular trafficking stresses (Figure 5D). Interestingly, the *grx4Δ* *cna1Δ* double mutants showed more severe growth defects than either of the single mutants on the agents that trigger ionic, osmotic and ER stresses, as revealed on medium with LiCl, NaCl, KCl, sorbitol, DTT, and tunicamycin (Figure 5D). However, the double mutants showed similar sensitivity as the *cna1Δ* single mutant to Ca^2+^, SDS, caffeine, Congo Red, caspofungin, and the trafficking inhibitor monensin (Figure 5D). In comparison, the *grx4Δ* mutants showed more robust growth on Ca^2+^, Congo Red, Caspofungin, and BFA. Together, these results support the idea that Grx4 may act in parallel with calcineurin to influence the responses to a subset of stress conditions (e.g., ionic, osmotic, and ER stress).

The calcineurin mutant, *cna1Δ*, displays elongated cells and multivesicular inclusions when grown in YPD at 37°C (Kozubowski *et al.* 2011). To examine the morphological phenotypes of the *grx4Δ* *cna1Δ* double mutants, we grew the cells in low iron medium and compared the morphologies with the single mutants and the WT strain. The *cna1Δ* mutant showed a similar morphology to WT cells, but exhibited elongated and clustered cells with multivesicular inclusions upon incubation in low iron medium for 16 hours (Figure 5E), as previously observed in YPD at 37°C (Cruz *et al.* 2001; Kozubowski *et al.* 2011). The cells of the *grx4Δ* mutant and a *crz1Δ* mutant (for comparison, see below) showed similar morphology to the WT cells at 30°C (Figure 5E). The defects for the *cna1Δ* mutant were more pronounced in the *grx4Δ* *cna1Δ* double mutants with enlarged or swollen cells and apparent defects in cell separation, thus suggesting an additional role for Grx4 in the regulation of cell morphology (Figure 5E).

### Grx4 and Crz1 have an additive influence on cell wall integrity and Grx4 influences the localization of Crz1

**Figure 6:**
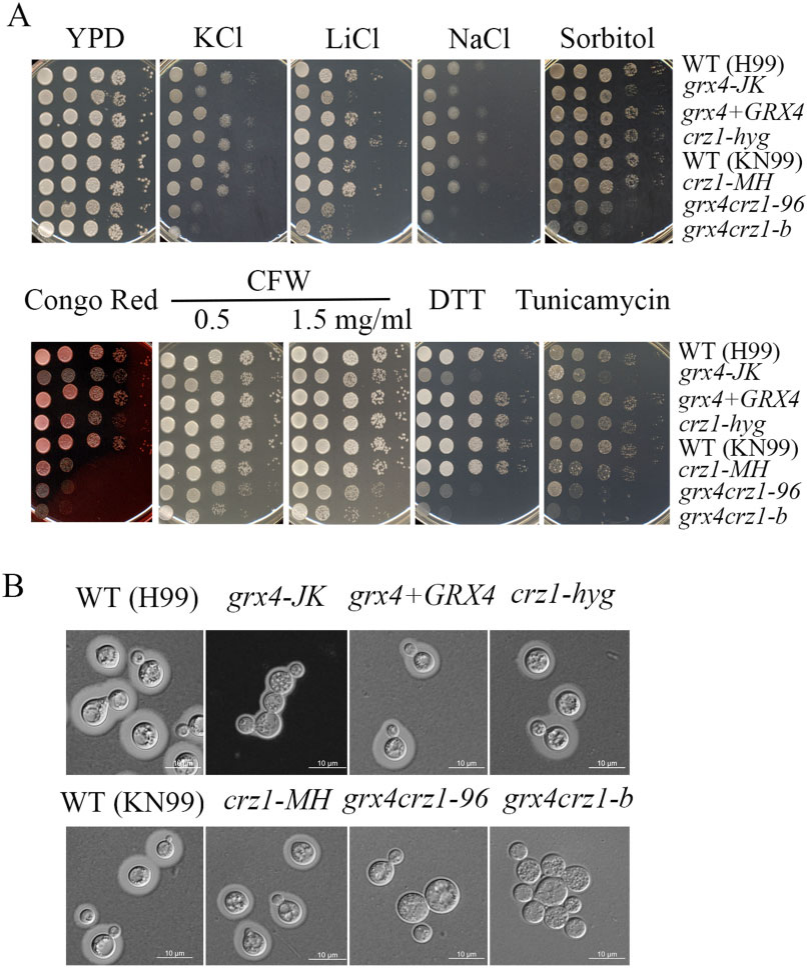
Grx4 interacts with Crz1 to regulate stress responses. (A) Cells of the indicated strains were grown overnight at 30°C, 10-fold serially diluted, and spotted on YPD supplemented with 1.5 M KCl, 75 mM LiCl, 1.5 M NaCl, 1M sorbitol, 0.5 mg/ml Congo Red, 0.65 mg/ml CFW, 15 mM DTT or 250 ng/ml tunicamycin. The two *crz1Δ* mutants are labeled *crz1-hyg* and *crz1-MH*, and the *grx4Δ crz1Δ* double deletion mutants are labeled *grx4 crz1-96* and *grx4 crz1-b.* The plates were incubated at 30°C for 2 days before being photographed. (B) To assess morphology and capsule formation, the cells were grown in defined low iron medium (capsule inducing medium) overnight at 30°C, stained with India ink, and examined under differential interference contrast microscopy. Bar = 10 µM.

In *S. cerevisiae* and *C. neoformans*, the transcription factor Crz1 is a major substrate of calcineurin, and Crz1 moves between the cytoplasm and the nucleus according to its activation state (Stathopoulos-Gerontides *et al.* 1999; Park *et al.* 2016; Chow *et al.* 2017). In *C. neoformans*, Chow *et al.* (2017) demonstrated that loss of Crz1 results in intermediate phenotypes compared to the WT strain and a *cna1Δ* mutant in response to stress conditions. We examined the interaction between Grx4 and Crz1 by comparing the phenotypes of the *grx4Δ* and *crz1Δ* single mutants and *grx4Δ* *crz1Δ* double mutants (Figure 6A). We employed a *crz1Δ* mutant (*crz1-hyg*) that we constructed in strain H99 as well as a mutant obtained from the deletion collection (*crz1-MH*) in the background strain KN99. In general, we found that the double mutant resembled the *grx4Δ* single mutant for phenotypes in response to ER, osmotic and ionic stresses, and CFW. The double mutant more closely resembled the *grx4Δ* mutant for growth on Congo Red, although we noted that the two *crz1Δ* mutants (*crz1-hyg* and *crz1-MH*) showed different sensitivities. We also assessed capsule formation in the mutants and found that the capsule defect of the *grx4Δ* mutant was shared by the double mutant (Figure 6B). Overall, these results indicated that Grx4 and Crz1 make a shared contribution to mediating susceptibility to specific stress conditions, although as noted below, Grx4 could function upstream of Crz1 through an influence on its subcellular localization.

**Figure 7:**
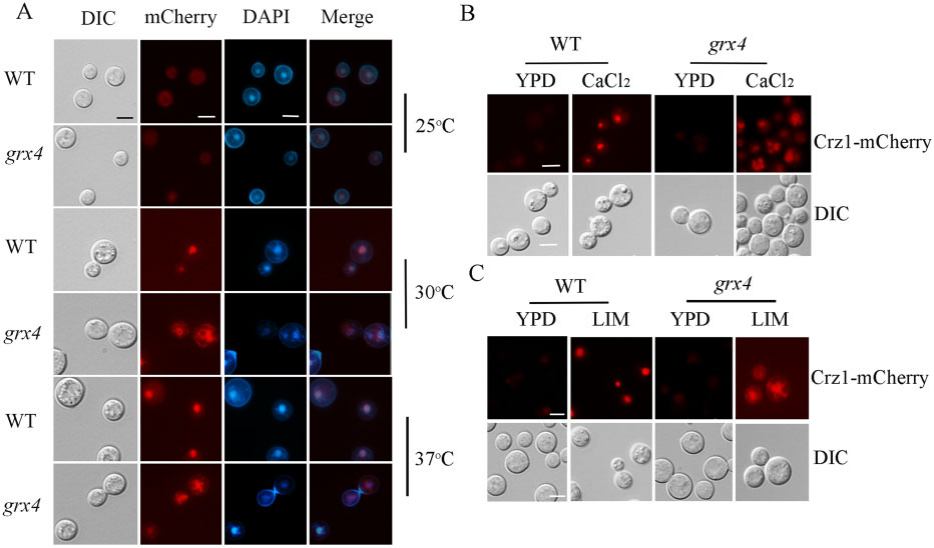
Deletion of *GRX4* influences localization of Crz1-mCherry. (A) Cells of the WT strain or the *grx4Δ* mutant (labeled *grx4*) expressing a Crz1-mCherry fusion protein were incubated in YPD overnight at the indicated temperatures, washed in PBS, and stained with DAPI (1 µg/ml, for staining nuclei) for staining nuclei) before microscopic observation. (B,C) Cells of the WT strain or the *grx4Δ* mutant expressing the Crz1-mCherry fusion protein were grown in YPD overnight at 25°C, washed and diluted in PBS. Cells were then transferred into fresh liquid YPD supplemented with or without 200 mM CaCl_2_ (B), or into low-iron YNB medium supplemented with 150 µM BPS (bathophenanthroline disulphonate, iron chelator) (C) and incubated at 25°C for 60 min before microscopic observation. Bar = 5 µM.

We next assessed the subcellular location of Crz1 to examine the connection between Crz1 and Grx4 in more detail. Translocation of Crz1 is an indicator of calcineurin activity in response to environmental stimuli (Stathopoulos-Gerontides *et al.* 1999; Lev *et al.* 2012; Chow *et al.* 2017). Following dephosphorylation by calcineurin, activated Crz1 translocates from the cytosol to the nucleus to trigger the expression of downstream genes. We examined the WT strain and the *grx4Δ* mutant expressing a Crz1-mCherry fusion protein and found that the weak fluorescent signal was diffuse and evenly distributed in the cytoplasm (Figure 7) in both backgrounds at 25°C. Upon incubation at 30°C or 37°C for 30 min, the Crz1-mCherry fluorescence signal was localized to the nuclei of WT cells as demonstrated by co-localization with DAPI (Figure 7A). We also noted that the Crz1-mCherry signal in the nucleus was more evident at 37°C. In contrast, loss of Grx4 altered the distribution of the Crz1-mCherry protein such that the signal was present both in the cytosol surrounding the nucleus and in nuclei (Figure 7A). In fungi, including *C. neoformans*, Crz1 also translocates from the cytosol to nucleus in response to extracellular calcium and glucosamine (Park *et al.* 2016; Chow *et al.* 2017; Xu *et al.* 2017). We therefore tested the influence of extracellular calcium on the Crz1-mCherry localization by addition of 400 mM calcium chloride to medium, and incubation at the lower temperature of 25°C for 60 min before observation (Figure 7B). The Crz1-mCherry fluorescence accumulated in nucleus after calcium addition in the WT strain, but the signal did not shift from the cytosol to nucleus in the *grx4Δ* mutant. In the mutant, Crz1-mCherry localized to large vesicular structures in the cytosol. We also examined the Crz1-mCherry localization in response to iron limitation and found that this condition triggered the nuclear location of Crz1 in the WT strain, but not in the *grx4Δ* mutant (Figure 7C). Crz1-mCherry fluorescence was mainly present in the cytosol surrounding nuclei in the mutant. Taken together, Grx4 influences the nuclear localization of Crz1 induced by elevated temperature, extracellular calcium, and iron limitation.

## Discussion

Monothiol glutaredoxins such as Grx4 are involved in iron regulation, and the biogenesis and trafficking of iron-sulfur clusters in a variety of fungi, including *A.* *fumigatus*, *C.* *albicans*, *C. neoformans*, *S. cerevisiae*, and *S. pombe* (Ojeda *et al.* 2006; Labbé *et al.* 2013; Encinar del Dedo *et al.* 2015; Dlouhy *et al.* 2017; Misslinger *et al.* 2019; Attarian *et al.* 2018; Alkafeef *et al.* 2020; Ramos-Alonso *et al.* 2020; Gupta and Outten 2020). We previously identified phenotypes for *grx4Δ* mutants related to iron homeostasis, including hypersensitivity to phleomycin, electron transport chain inhibitors, and agents that provoke oxidative stress and DNA damage (Attarian *et al.* 2018). Loss of Grx4 also eliminates the ability of *C. neoformans* to cause disease in a mouse model of cryptococcosis. In this study, we discovered that Grx4 had a broader contribution to stress resistance including thermotolerance, a critical attribute for pathogenic fungi to cause disease in mammalian hosts. Notably, the impaired thermotolerance of a *grx4Δ* mutant may partially explain the avirulence of the *grx4Δ* mutant (Attarian *et al.* 2018). Consistent with our observations, the Grx4 ortholog in another human pathogenic fungus, *C. albicans*, is also required for robust growth at elevated temperature indicating a conserved role in thermotolerance (Alkafeef *et al.* 2020). Other phenotypes of the *grx4Δ* mutant in *C. neoformans* may also be important in the context of disease and identification of therapeutic targets. These traits include hypersensitivity to agents that challenge membrane and CWI, ionic and osmotic stress, agents that challenge ER function, and trafficking inhibitors. Consistent with this idea, we found sensitivity to fluconazole and amphotericin B that target membrane-related functions and that are frontline antifungal drugs for treating cryptococcosis.

**Figure 8:**
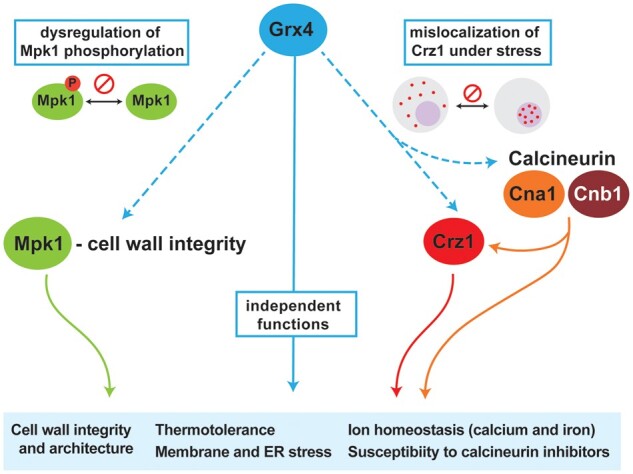
Summary of the regulatory influences of Grx4 on the responses to stress mediated by Mpk1, Crz1, and calcineurin. The diagram highlights the influence of Grx4 on traits related to virulence (*e.g.*, thermotolerance, cell wall architecture, membrane and ER stress, and ion homeostasis). These traits are shown connected to the cell wall integrity pathway through an influence of Grx4 on Mpk1 phosphorylation. Connections to shared phenotypes with mutants defective in calcineurin signaling are depicted including an influence on the nuclear localization of the transcription factor Crz1.

A key outcome of our study is that Grx4 has emerged as an important link between iron homeostasis and the variety of stress responses outlined above (Figure 8). We previously found that Grx4 interacts with Cir1, the iron-responsive transcription factor that regulates iron uptake and utilization and virulence in *C. neoformans* (Attarian *et al.* 2018). Our current findings are consistent with our analysis of *cir1Δ* mutants that demonstrated a variety of phenotypes including impaired growth at 37°C, as well as sensitivity to FK506, elevated calcium, SDS, amphotericin B, and miconazole (Jung *et al.* 2006). The *grx4Δ* and *cir1Δ* mutants share these phenotypes and both proteins influence the formation of major virulence factors. In addition to iron sensing, Cir1 also regulates components of the CWI at the transcriptional level (Jung *et al.* 2006). The interaction of Grx4 with Cir1 provides one example to explain the multitude of phenotypic contributions for Grx4 in that the protein may interact with a number of transcription factors and other regulators to influence multiple functions. The role of monothiol glutaredoxins via interactions with transcription factors appears to be conserved because Grx3 and Grx4 in *S. cerevisiae* also function in iron homeostasis by regulating the iron-responsive transcription factor Aft1 (Gupta and Outten 2020), and participate in the defense against oxidative stress caused by reactive oxygen species (ROS) (Pujol-Carrion and Torre-Ruiz 2017). Furthermore, Grx4 also plays a role in oxidative stress response in *C. albicans* (Alkafeef *et al.* 2020). Notably, the iron regulator SreA in *Alternaria alternata* that is similar to Cir1 also influences sensitivity to oxidative stress and agents that challenge CWI (Chung *et al.* 2020). In addition, a recent study revealed that the MAPK Slt2/Mpk1 regulates the iron regulatory transcription factor Atf1 to influence iron homeostasis in *S. cerevisiae* (Pujol-Carrion *et al.* 2021). This finding suggests that the linkages between regulatory factors that control iron homeostasis and stress responses may be conserved among fungal pathogens.

The multiple stress-related phenotypes of the *grx4Δ* mutant suggested a potential interaction of Grx4 with well-characterized stress response pathways, and we identified shared phenotypes with mutants lacking calcineurin subunits, the calcineurin target Crz1, and the CWI pathway MAPK Mpk1 (Kraus *et al.* 2003; Gerik *et al.* 2005, 2008; Wang *et al.* 2011; Lam *et al.* 2013; Park *et al.* 2016, 2019; Chow *et al.* 2017; Fu *et al.* 2018; Jiménez-Gutiérrez *et al.* 2020). Several lines of evidence suggested that Grx4 may influence thermotolerance in parallel with calcineurin signaling including impaired growth in limited or excess calcium, hypersensitivity to calcineurin-specific inhibitors, and mis-localization of the calcineurin substrate Crz1. As a conserved stress regulator, calcineurin regulates multiple processes including morphogenesis, mating, CWI and virulence, and is a potential drug target in pathogenic fungi and parasitic protists (Yoshida *et al.* 1994; Fox *et al.* 2003; Fox and Heitman 2005; Chow *et al.* 2017; Fu *et al.* 2018; Park *et al.* 2019; Juvvadi *et al.* 2020). The potential connection between Grx4 and calcineurin signaling has not been explored in pathogenic fungi, and our analysis suggests parallel activities, and possible genetic interactions, that contribute to the response to stress (Figure 8). This conclusion is based on the finding that the *grx4Δ* *cna1Δ* double mutant that exhibited greater sensitivity to some conditions (e.g., ionic, osmotic, and ER stress) but not others (e.g., treatment with Ca^2+^, Congo Red, CFW, BFA) compared to the single *cna1Δ* or *grx4Δ* mutants. Loss of the calcineurin subunit Cna1 in *C. neoformans* causes a slightly elongated and a multivesicular cell morphology at 37°C, and loss of *GRX4* in the *cna1Δ* background (*grx4Δ* *cna1Δ*) results in more significant change in morphology in response to iron limitation, suggesting that Grx4 is also involved in determining cell morphology under nutrient stress. Monothiol glutaredoxins influence the organization of the actin cytoskeleton in *S. cerevisiae* and this role is important for the ability of cells to respond to oxidative stress (Pujol-Carrion and de la Torre-Ruiz 2010, Pujol-Carrion *et al.* 2013). It is also known that calcineurin is a metalloenzyme that contains Fe and Zn in addition to binding other metals (King and Huang 1984). In this context, we speculate that iron homeostasis mediated by Grx4, in association with key transcriptional regulators such as Cir1, may influence calcineurin activity due to metal availability as well as potentially by transcriptional control (Jung *et al.* 2006).

Chow *et al.* (2017) demonstrated that calcineurin regulates the transcription of genes via Crz1 to influence CWI, survival under stress conditions, and virulence. In addition, calcineurin regulates the transcription of other functions and influences temperature sensitivity independent of Crz1 (Chow *et al.* 2017). We found that *grx4Δ* *crz1Δ* double mutants had phenotypes that more closely resembled those of the *grx4Δ* single mutant thus raising the possibility of a genetic interaction, an idea supported by the finding that Crz1 localization is perturbed in a *grx4Δ* mutant. We propose therefore that part of the Grx4 contribution to stress responses occurs via an influence on Crz1 localization to the nucleus to regulate transcription of genes for stress resistance (Figure 8).

We were also intrigued by the role of Grx4 in supporting cell wall and membrane integrity, and a clue to the underlying mechanism came from the finding that the Slt2 MAPK of the CWI pathway in *S. cerevisiae* physically interacts with two redundant monothiol glutaredoxins Grx3 and Grx4 in response to oxidative stress (Pujol-Carrion *et al.* 2017). The Slt2 ortholog Mpk1 has been well characterized in *C. neoformans* and loss of the protein causes a similar set of phenotypes as loss of Grx4 including increased sensitivity to elevated temperature, as well as membrane, cell wall, and ionic stresses (Kraus *et al.* 2003; Gerik *et al.* 2005, 2008; Wang *et al.* 2011; Lam *et al.* 2013; Yang *et al.* 2017). Several studies have demonstrated that the phosphorylation state of Mpk1 is responsive to a variety of stresses (e.g., elevated temperature, treatment with SDS or CFW) in *C. neoformans*, and these observations echo the findings in *S. cerevisiae* (Kraus *et al.* 2003; Gerik *et al.* 2008; Wang *et al.* 2011; Lam *et al.* 2013; Jiménez-Gutiérrez *et al.* 2020). The Mpk1 signaling pathway also contributes to the virulence of *C. neoformans* (Gerik *et al.* 2008). Our analysis revealed that the level of Mpk1 phosphorylation is reduced and delayed in the *grx4Δ* mutant upon exposure to elevated temperature or cell wall stresses. Interestingly, treatment with SDS resulted in elevated Mpk1 phosphorylation in the *grx4Δ* mutant suggesting a distinct influence compared with the other stress conditions. In addition to provoking membrane and cell wall stress, SDS is known to also induce oxidative stress (Cao *et al.* 2020). Therefore, SDS may activate Mpk1 by mechanisms that bypass Grx4. Although a physical interaction between Grx4 and Slt2 (Mpk1) has been demonstrated in *S. cerevisiae* by the yeast two hybrid assay (Pujol-Carrion and Torre-Ruiz 2017), we were unable to detect an interaction between Grx4 and Mpk1 by the same approach. Confounding factors may include the requirement for specific stress conditions or additional interacting partners, and/or the presences of a transient or weak interaction in *C. neoformans*. We also note that a preliminary affinity purification-mass spectrometry experiment in *C. neoformans* did not reveal an interaction between Grx4 and Mpk1, although the known interaction between Grx4 and Cir1 was detected (Attarian *et al.* 2018; G. H., unpublished results). Therefore, the mechanism by which Grx4 influences Mpk1 phosphorylation remains to be determined and a first step will be to examine a possible interaction between Cir1 and Mpk1. We did observe an increased abundance of Mpk1 protein, but not *MPK1* transcripts, in the *grx4Δ* mutant suggesting a post-transcriptional influence of Grx4. Overall, we propose that, along with an influence on Crz1 localization, the contribution of Grx4 to stress resistance is due to an influence on the CWI pathway, as indicated by the shared phenotypes of *grx4Δ* and *mpk1Δ* mutants and the influence of Grx4 on Mpk1 phosphorylation. This influence likely accounts for the observed changes in cell wall composition in the *grx4Δ* mutant.

In summary, the monothiol glutaredoxin Grx4 influences a variety of cell processes essential for fungal virulence, including iron sensing, thermotolerance, stress responses, and CWI in *C. neoformans*. These functions result in part from an influence on the Pkc1-Mpk1 signaling pathway and the localization of Crz1, as well as a parallel interaction with calcineurin signaling.

## Data availability

Strains and plasmids are available upon request and are listed in Supplementary Table S1. Primers used to generate mutants and tagging strains, and Supplementary Table S2. Primers used in qRT-PCR. The authors affirm that all data necessary for confirming the conclusions of the article are present within the article, figures, and tables.

Supplementary material is available at *G3* online.

## Supplementary Material

jkab322_Supplementary_Data
