# Supplementary material for: The monothiol glutaredoxin Grx4 influences thermotolerance, cell wall integrity, and Mpk1 signaling in Cryptococcus neoformans
Source: G3 (Bethesda). 2021 Sep 7;11(11):jkab322. doi: 10.1093/g3journal/jkab322 (PMC8527476; doi:10.1093/g3journal/jkab322)
Supplement: jkab322_Supplementary_Data [file jkab322_supplementary_data.pdf]

Supplemental Table S1. Primers used to generate mutants and tagging strains

Primer sequence (5' – 3')	Primer name
TCGGGCTACGTTTGGAATTAGCCTCTCCT	Cna1-del-P1
GCCTCTGCGTCTTCGCCCACTCCTACCACCAGGTTTATCTGTATTAACACGGAAG	Cna1-del-P2
CTTCCGTGTTAATACAGATAAACCTGGTGGTAGGAGTGGGCGAAGACGCAGAGGC	Cna1-del-P3
CCAGCTCACATCCTCGCAGCGAGTAGATGCTGGTTTCGAACGATGGA	Cna1-del-P4
TCCATCGTTCGAAACCAGCATCTACTCGCTGCGAGGATGTGAGCTGG	Cna1-del-P5
CAAGACCCTGGCTACTGTTGTAACTTACCCT	Cna1-del-P6
GGCAGAAACTGACATGCGACACAGA	Cna1-del-P7-NE
GAGATAACGAGTATCTGCCGGAT	Cna1-del-P8-NE
CTCCCAATCTGCTGGATGACATCT	Cna1-del-P9-PO
TGTCTGGGAGTCTTTCTGGATTGGAGT	Cna1-P10-PO
TGCCGATTGCGAGCTCAAGATTTGTGATT	Mpk1-Flag-P1F
GCTGTGGTCCAAGTCTGATAATAGATCTAGACA	Mpk1-Flag-P3R

Supplemental Table S2. Primers used in qRT-PCR

Primer sequence (5' – 3')	Primer name
GTATCCTAACGCATCTCCCTTG	Mpk1For
GGAAACTTCTGACCTCTTCGAG	Mpk1Rev
AACAGGTCTGTGATGCCCTTAGA	18sFor
ACTCGCTGGCTCAGTCAGTGT	18sRev
CCACACTGTCCCCATTTACGA	ACT1For
CAGCAAGATCGATACGGAGGAT	ACT1Rev
